# Retrograde Intubation in a Severe Fixed Spinal Deformity: A Case Report

**DOI:** 10.5811/cpcem.50583

**Published:** 2026-06-26

**Authors:** Shrirang Joshi, Shreesha Nayak, Soubhik Chakraborty, Hardeep Kaur, Naazia Siddiqua, Sanjeev Bhoi

**Affiliations:** All India Institute of Medical Sciences Delhi, Department of Emergency Medicine, New Delhi, India

**Keywords:** retrograde intubation, difficult airway, ankylosing spondylitis, kyphosis, emergency medicine

## Abstract

**Introduction:**

Retrograde intubation is a seldom used but valuable technique in managing difficult airways, especially in resource-limited settings when advanced equipment is unavailable or ineffective.

**Case Report:**

We report the case of a 64-year-old male with advanced ankylosing spondylitis and severe cervicothoracolumbar kyphosis who presented with altered mental status and respiratory distress. Extreme fixed cervical flexion, markedly restricted mouth opening, and an inaccessible anterior neck rendered direct laryngoscopy, video laryngoscopy, fiberoptic intubation, and surgical airway approaches unfeasible. Retrograde nasotracheal intubation was successfully performed, resulting in airway stabilization and hemodynamic improvement.

**Conclusion:**

This case highlights retrograde intubation as a lifesaving low-technology technique in complex anatomic and physiologic airways, demonstrating its continued relevance under the updated Difficult Airway Society 2025 guidelines.

## INTRODUCTION

Airway management is fundamental to emergency medicine, as failure to secure the airway promptly can lead to hypoxia, neurologic injury, or death. The risk of complications increases significantly in patients with distorted airway anatomy or physiologic instability, where conventional techniques may fail. Modern guidelines, including the updated Difficult Airway Society 2025 recommendations, emphasize maintaining multiple fallback airway strategies—particularly low-technology rescue techniques—when advanced tools are unavailable or ineffective.^1^,^2^

Retrograde intubation has been successfully used in cases involving restricted mouth opening, temporomandibular joint ankylosis, oral submucosal fibrosis, craniofacial anomalies, cervical spine immobility, and bleeding upper airways.^3^–^5^ Despite being well established, this technique is underused in contemporary emergency practice where clinicians increasingly rely on video laryngoscopy and fiberoptic bronchoscopy. This case illustrates the critical value of retrograde nasotracheal intubation in a patient with extreme fixed spinal deformity, physiologic instability, and complete loss of surgically accessible neck landmarks—highlighting that retrograde intubation remains an essential rescue technique in modern emergency medicine.

## CASE REPORT

A 64-year-old male with prior middle cerebral artery stroke and advanced ankylosing spondylitis with severe cervicothoracolumbar kyphosis presented with decreased responsiveness for four hours following vomiting. Initial vital signs were as follows: heart rate, 148 beats per minute; respiratory rate, 36 breaths per minute with bilateral coarse crackles; blood pressure, 60 millimeters of mercury (mm Hg) systolic (improving to 90/58 mm Hg after a bolus of one liter of Ringer’s lactate); and oxygen saturation, 68% on room air (improving to 88% with a non-rebreather mask). Glasgow Coma Scale (GCS) was 7 (Eye-1 Verbal-1 Motor-5). Pupils were 3 millimeters (mm) and reactive.

Blood glucose was 138 milligrams per deciliter (mg/dL) (reference range: 70–140 mg/dL).

Arterial blood gas analysis showed pH, 7.49 (7.35–7.45); partial pressure of carbon dioxide, 25.3 mm Hg (35–45 mm Hg); bicarbonate, 19.2 millimoles per liter (mmol/L) (22–26 mmol/L); and lactate, 6.1 mmol/L (< 2 mmol/L), consistent with respiratory alkalosis and metabolic acidosis. Immediate intubation was indicated.

Airway evaluation revealed severe fixed cervical flexion preventing alignment of airway axes, restricted mouth opening (two fingerbreadths), and extreme kyphosis that prevented the patient from lying supine. Partial positioning was achieved by elevating the legs and stabilizing the torso with gurney side rails. Direct laryngoscopy and video laryngoscopy were impossible due to the degree of deformity. Two attempts at blind nasotracheal intubation failed. The anterior neck was deeply obscured beneath the sharply flexed cervicothoracic curve, and the thyroid cartilage lay below the sternal notch, making surgical airway interventions—including cricothyrotomy and tracheostomy—anatomically inaccessible.

Due to the absence of fiberoptic bronchoscopy, retrograde nasotracheal intubation was performed. A 16-gauge needle was inserted through the cricothyroid membrane during inspiratory elevation. A 150-cm flexible guidewire was advanced cephalad and retrieved from the right nostril. A 7.0-mm endotracheal tube was railroaded over the guidewire and advanced until appropriate resistance was felt. After withdrawing the guidewire and needle, bilateral breath sounds and continuous end-tidal carbon dioxide confirmed tube placement. The tube was secured at 28 cm, which was appropriate for the patient’s extreme cervical flexion. A chest radiograph confirmed mid-tracheal position approximately 3 cm above the carina.

The patient tolerated the procedure well and remained hemodynamically stable following intubation (Image).

*CPC-EM Capsule*

What do we already know about this clinical entity?

*Retrograde intubation is an established rescue airway technique when conventional and advanced intubation methods fail.*

What makes this presentation of disease reportable?

*Extreme fixed spinal deformity eliminated all standard and surgical airway options, leaving retrograde intubation as the only viable approach.*

What is the major learning point?

*Retrograde intubation remains a critical low-technology rescue skill in anatomically extreme and physiologically unstable airways.*

How might this improve emergency medicine practice?

*This case reinforces the need to be prepared for airway device failure and the continued relevance of foundational rescue airway techniques.*

## DISCUSSION

This case demonstrates a uniquely complex airway due to profound ankylosing spondylitis–related deformity, fixed cervical flexion, physiologic instability, and complete inaccessibility of anterior neck landmarks. Such scenarios are rarely reported in the literature, especially those requiring retrograde nasotracheal intubation. The most recent published research (2020–2025) reinforces that retrograde intubation remains an important rescue technique when video laryngoscopy and fiberoptic devices fail, are unavailable, or are rendered ineffective by blood, secretions, or distorted anatomy.^3^,^4^ Retrograde intubation requires minimal equipment, is inexpensive, and maintains effectiveness even in physiologically unstable patients.

Fiberoptic intubation, although a gold standard for many predicted difficult airways, requires functioning equipment, patient stability, and operator expertise—limitations often encountered in resource-poor emergency situations.^6^ Retrograde intubation, therefore, complements rather than competes with modern airway tools. Ultrasound guidance has improved identification of airway landmarks, particularly in obese or anatomically distorted patients, expanding safe application of retrograde techniques.^5^ The Dificult Airway Society 2025 guidelines emphasize retention of such foundational, low-technology skills, in addition to video laryngoscopy and fiberoptic methods, advocating preparedness for failure of first-line approaches.^1^,^6^

## CONCLUSION

Emergency physicians frequently encounter patients without prior airway assessment and manage a large proportion of trauma airways. Maintaining competence in retrograde intubation ensures readiness when modern devices or surgical airways are not viable options. This case serves as a reminder that even today, in the era of video laryngoscopy and fiberoptic scopes, retrograde intubation remains a life-saving technique in resource-limited and anatomically extreme scenarios.

## Figures and Tables

**Image.**
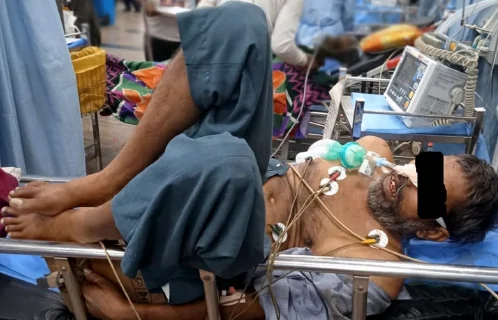
Clinical photograph showing severe cervicothoracolumbar kyphosis and fixed cervical flexion, resulting in the failure of conventional intubation techniques and the necessity of retrograde intubation.

## References

[1] Ahmad I, El-Boghdadly K, Iliff H (2026). Difficult Airway Society (DAS) 2025 guidelines for management of unanticipated difficult tracheal intubation in adults. Br J Anaesth.

[2] Apfelbaum JL, Hagberg CA, Connis RT (2022). 2022 American Society of Anesthesiologists practice guidelines for management of the difficult airway. Anesthesiology.

[3] Tiwari T, Sharma B, Rajput SK (2022). Retrograde intubation as a rescue procedure in unanticipated difficult airway: an old technique still relevant. Med Gas Res.

[4] Tiwari T, Walian A, Singh VK (2020). Evaluation of retrograde intubation with different doses of dexmedetomidine infusion: a randomized controlled trial. J Oral Biol Craniofac Res.

[5] Vieira D, Lages N, Dias J (2013). Ultrasound-guided retrograde intubation. Anaesthesia.

[6] Dunford B, Sutterfield B, Roberts W (2025). Difficult airway management: an analysis of systematic review evidence underpinning clinical practice guidelines. Anaesth Crit Care Pain Med.

